# Contralateral facial artery myomucosal island flap for the reconstruction of T_2_-T_3_ oncologic oral defects

**DOI:** 10.3389/fonc.2024.1393687

**Published:** 2024-06-04

**Authors:** Min Huang, Peiyao Li, Le Yang, Yudong Xiao, Lingchan Zeng, Yuxiong Su, Yujie Liang, Gucheng Zeng, Guiqing Liao, Sien Zhang

**Affiliations:** ^1^ Department of Oral and Maxillofacial Surgery, Hospital of Stomatology, Guanghua School of Stomatology, Sun Yat-sen University, Guangzhou, China; ^2^ Department of Medical Records, Hospital of Stomatology, Guanghua School of Stomatology, Sun Yat-sen University, Guangzhou, China; ^3^ Division of Oral and Maxillofacial Surgery, Faculty of Dentistry, The University of Hong Kong, Hong Kong, Hong Kong SAR, China; ^4^ Department of Microbiology, Zhongshan School of Medicine, Key Laboratory for Tropical Diseases Control of the Ministry of Education, Sun Yat-sen University, Guangzhou, China

**Keywords:** oral cancer, head and neck cancer, tongue cancer, reconstructive surgery, plastic surgery, pedicled flap

## Abstract

**Objectives:**

To avoid the oncologic risks of ipsilateral regional flaps, this study aimed to explore the feasibility and clinical outcomes of the contralateral-based facial artery myomucosal island flap (C-FAMMIF) for oral T_2_–T_3_ oncologic defects reconstruction.

**Methods:**

A study of flap anatomy was conducted on 7 cadaver samples and a cohort of 24 patients who received C-FAMMIF reconstruction after malignancy resection were retrospectively researched. A balanced anterolateral thigh flap (ALT) group of 47 patients was extracted as control group using propensity score matching method. Progression-free survival (PFS), functional outcomes, and donor site complications were assessed.

**Results:**

Consistent blood supply and drainage through facial artery and vein with median maximum pedicle length of 106 mm supported contralateral reconstruction. The superficial vein drainage pattern indicated safer flap harvest at contralateral neck under circumstances of ipsilateral neck dissections. The pedicle and marginal facial nerve formed three anatomical patterns. The surgical management of each was described. Patients with ipsilateral pN_+_ neck accounted for 41.7% and 40.4% in the C-FAMMIF and ALT group, respectively. The 2-year PFS rate between the C-FAMMIF and ALT groups was not significantly different (88.2% in C-FAMMIF group and 84.6% in ALT group, respectively, *p* = 0.6358). Promising recoveries were observed for swallowing function and tactile sensation. The donor sites healed upon primary closure without trismus or permanent facial palsy.

**Conclusion:**

Our findings suggested that C-FAMMIF is feasible and safe for T_2_-T_3_ oral oncologic defect reconstruction in patients with ipsilateral cN_+_ neck.

## 1 Introduction

Oral maxillofacial tumor resection results in various tissue defects requiring immediate reconstruction. Filling the dead space and covering the wound are the basic needs; however, the ultimate goal of reconstruction is to rehabilitate both the contour and function, which determines the requirements for the flap to be used. It should possess proper bulk and texture, which are consistent with the defective structure, be easy to harvest, and most importantly, be oncologically safe (1). The oral cavity is a damp, sensitive, and delicate space that requires smooth and flexible restoration to maintain physical functions such as talking, swallowing, and feeling. The buccal mucosa, the floor of mouth (FOM), and tongue tip are particular sites requiring thin, pliable flaps with smooth surfaces (2), especially in T_2_–T_3_ staged patients with only small-to-medium-sized soft tissue defects.

In oral defect reconstruction, the drawbacks associated with the adoption of cutaneous flaps to replace the original mucosal tissue outstand: (1) Being too thick in obese patients; (3, 4) (2) Bearing hair that feels uncomfortable in the oral cavity; (3) Developing chronic inflammation to the point of secondary carcinoma because of the constant saliva stimulation; (5–8) and (4) Shrinkage of the skin paddle resulting in trismus and tongue inflexibility (9, 10).

As put forward by Doctor Harold Gilies, the principle of plastic surgery is “losses must be replaced in kind” (11). The buccal mucosa is an ideal donor site of mucosal tissue, and the buccinator and masseter muscles can provide sufficient tissue bulk. The first buccal myomucosal flap was described in 1992 by Dr Pribaz et al. as the facial artery myomucosal flap (FAMM) (12); however, it was locally designed without dissecting the blood vessels. Thus, the rotation diameter was limited, and sometimes, a secondary surgery was needed to cut the pedicle (13). In 1999, Dr Zhao’s team modified this flap to an island flap that could be tunneled, hence gaining a larger restoration range (14). Notably, the island myomucosal flap based on the facial artery turned out to be very effective in small-to-medium oral defect reconstruction with good functional and cosmetic outcomes (15, 16).

However, oncological safety must be considered first. Oral cancer, especially squamous cell carcinoma (SCC), is likely to spread through the lymphatic system (17). Lymph nodes consistently exist around the submandibular gland and are closely related to the facial artery and vein. It has been documented that 90.7% of the level I lymph nodes received drainage from the tongue, gingiva, and cheek (18). This level, which is located by the flap pedicle, is the most common region of lymphatic metastasis from oral cancer, accounting for 20% in the cN_0_ necks and 48% in the cN_+_ necks (18, 19). The locoregional recurrence rate related to the ipsilateral submental island flap transfer was reported to be 19.05% in patients with positive lymph nodes in level I (20). Careful skeletonization of the flap pedicle was recommended to avoid tumor dissemination; however, it increases the risks of vascular crisis (18). Preserving the facial blood vessels and flap during neck dissection impedes surgical radicalness.

The application of contralateral facial artery myomucosal flap (C-FAMMIF) in the reconstruction of oral defects was rarely researched in previous literature. This study aimed to explore the feasibility and clinical outcomes of the C-FAMMIF for small-to-medium oncologic oral defect reconstruction through anatomical research and a retrospective study of 24 patients.

## 2 Materials and methods

### 2.1 Ethical approval

The present study was approved by the ethics committee of Guanghua School of Stomatology, Sun Yat-sen University.

### 2.2 Patient enrollment and propensity score matching

All practices in this study complied with the Hippocratic oath and were approved by the ethics committee. The 24 patients enrolled in this study met the following inclusion criteria: (1) surgical treatment between May 2020 and October 2022 in the Department of Oral and Maxillofacial Surgery, Hospital of Stomatology, Sun Yat-sen University; (2) pathological diagnosis of oral SCC (OSCC); (3) T_2_–T_3_ staged tumor according to the American Joint Committee on Cancer (AJCC) Cancer Staging Manual (8^th^ Edition); and (4) use of C-FAMMIF for oncologic defect reconstruction. Patients who met one of the following criteria were excluded: (1) history of tumor relapse and (2) inaccessible records or loss to follow-up.

To evaluate the oncological safety of C-FAMMIF, the most frequently applied distal flap, the anterolateral thigh flap (ALT), was selected as the control group. Propensity score matching (PSM) was conducted to obtain a baseline-balanced ALT group with adjustments for age, sex, tumor site, tumor TNM (topography, lymph node and metastasis) stage, and neck dissection, which were considered to influence both flap selection and survival outcomes. The nearest-neighbor matching method with 0.05 calliper and a 1:2 ratio was utilized to extract a 48-patient-size ALT group, among whom one patient was lost to follow-up and excluded. Eventually 47 patients were enrolled in the ALT group. The Kernal density distribution plot of the propensity score and Pearson χ^2^ test were used to check the baseline data balance.

### 2.3 Cadaver dissection

Pigmented liquid latex was injected into seven cadaver samples to mark the arteries and veins. Dissection focused on the facial arteries, veins, and their branches. The number and diameter of the buccal and masseter branches and maximum pedicle lengths (from the buccal branch origin to the facial artery origin) were measured.

### 2.4 Surgical method

**Figure 1:**
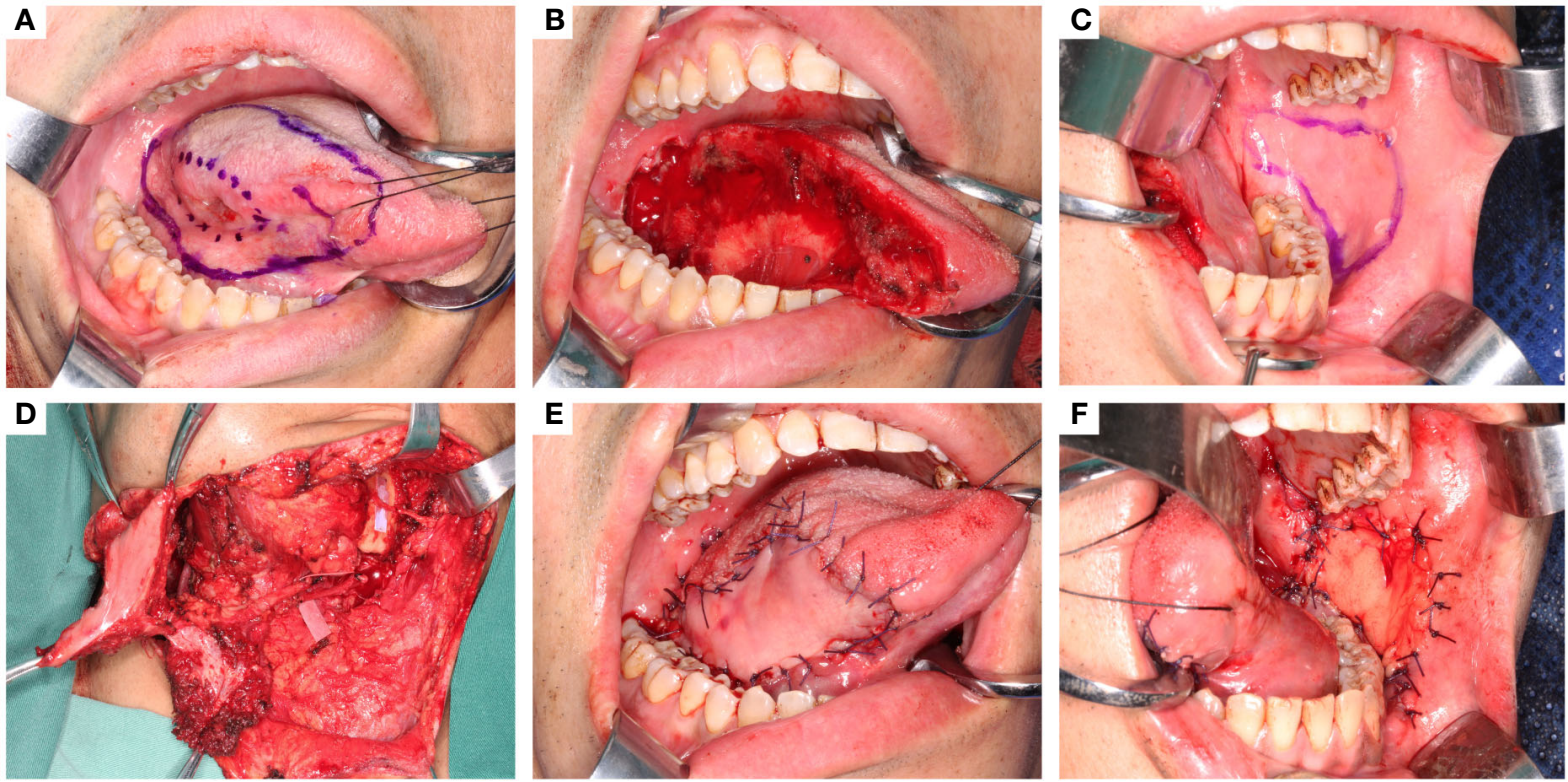
The oncologic tongue defect reconstruction using C-FAMMIF. **(A)** tumor appearance and the resection range. **(B)** the defect after tumor resection. **(C)** design of the C-BMMIF. **(D)** harvest of the C-FAMMIF. **(E)** primary tongue reconstruction with C-BMMIF. **(F)** donor site closure with buccal fat pad.

The tongue cancer appearance and the defect after tumor ablation are shown in Figures A and B. The flap contour was drawn on the contralateral buccal mucosa, with the superior border at least 5 mm below the parotid duct orifice and the anterior border at least 10 mm from the oral commissure; the other borders extended posteriorly to the pterygomandibular ligament and the mandibular vestibule inferiorly, depending on the defect size. This shape is designed along the facial artery (**Figure 1C**). In our practice, the largest flap measured 70 × 50 mm without parotid duct translocation.

The contralateral facial artery was located before the contralateral submandibular incision was made. After elevating the platysma, the marginal branch of the facial nerve was carefully identified and protected. The pedicle was dissected superiorly towards the buccal muscle, and the branches were separated and ligated. The masseter branch is consistent with the facial artery. If the defect was more than 1/3 of the tongue, the masseter artery was preserved, and a masseter muscle island was included in the flap (**Figure 1D**). The dissection layer was superficial to the facial artery and vein to ensure that the blood vessels were on the flap side. An appropriate amount of soft tissue was attached to the pedicle to avoid vasospasms. The pedicle was within the designed flap contour before the flap was cut and elevated from the intraoral side. Subsequently, the distal ends of the facial vessels were ligated and cut. The flap was raised and tunneled through the external mandibular tunnel to the neck (**Figure 1D**) and then through the internal mandibular tunnel into the oral cavity (**Figure 1E**). The buccal donor site was closed by pulling and suturing the buccal fat pads (**Figure 1F**).

During the surgical procedure, the venous drainage path and pedicle-nerve relationship were recorded.

### 2.5 Patient assessment

Patients were assessed before surgery, 1 month after surgery, and every 3 months thereafter. PFS, functional outcomes (swallowing and sensation), and donor-site conditions (mouth opening and facial nerve function) were evaluated.

Water swallowing test (WST): Patients were required to sit upright and drink 30 ml of water as quickly as possible. We graded the dysphagia from I–V depending on swallowing efficacy and the extent of choking: level I, patient swallowed in one gulp without choking; level II, patient swallowed in several gulps without choking; level III, patient swallowed in one gulp with moderate choking; level IV, patient swallowed in several gulps with moderate choking; and level V, patient experienced severe choking and was unable to finish 30 ml water.

Semmes-Weinstein monofilament test: Standardized Semmes-Weinstein monofilament tools (Premier Products, USA, consisting of a series of filaments with gradient diameters) were used. Filaments were applied perpendicularly to the flap mucosa until they were slightly bent for 1.5 s, in ascending order, until the patient felt the pressure. The stimulation thresholds were recorded.

The House-Brackman Facial Paralysis Scale (**Supplementary Table 1**) was used by two qualified doctors to independently evaluate facial nerve function.

Pre- and postsurgical smiling photos were analyzed to assess facial symmetry. The smile angle was determined between the midline and the line connecting the angulus oris and the lower lip midpoint. (**Supplementary Figure 1**).

### 2.6 Statistical analyses

All data were analyzed using the Statistical Package for Social Sciences (IBM SPSS Statistics 25, USA). The baseline information of the two groups were compared with the Pearson χ^2^ test. The PFS was measured from the date of treatment to the event of relapse, metastasis, or death. Log-rank tests and Kaplan-Meier plots were used to compare the survival outcomes of the two groups. The t-test was used for continuous data, which followed a normal distribution, whereas the smile angle on both sides was analyzed using a paired t-test. One-way analysis of variance (ANOVA) followed by Tukey’s honestly significant difference (HSD) paired comparisons were employed to compare inter-group differences when the data followed a normal distribution. Categorical data and data that did not follow a normal distribution were analyzed using the Kruskal–Wallis H test with Bonferroni correction.

## 3 Results

### 3.1 Blood supply and pedicle anatomy of the C-FAMMIF

**Figure 2:**
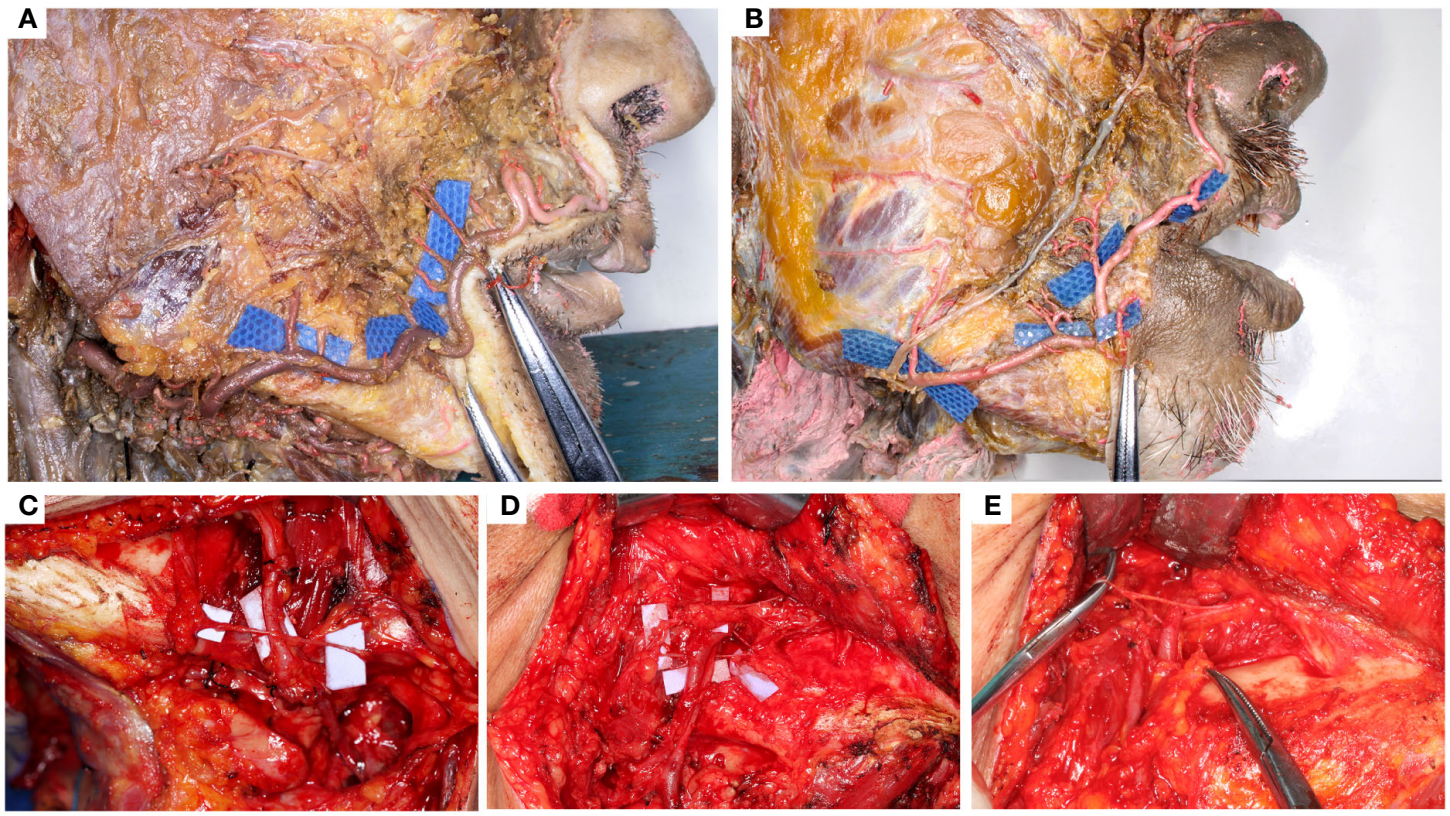
The blood supply from facial artery and nerve pedicle patterns. **(A, B)** the two patterns of the buccal branches. **(A)** type I: multiple independent buccal branches directly originate from the facial artery; **(B)** Type II: a few trunks give out dentritic buccal branches. **(C–E)** the three patterns of the pedicle-nerve relationships. **(C)** type I: the marginal facial nerve was superficial to the facial vessels; **(D)** type II: the marginal facial nerve circled the facial vessels; **(E)** type III: the marginal facial nerve was trapped between the facial vessels.

The existence of buccal branches and the masseter artery was stable, with a median diameter of 0.50 (0.20–1.50) mm and 0.90 (0.50–1.20) mm, respectively (**Table 1**). The facial artery supplied the buccal muscles in two patterns. Three of the seven samples had buccal branches directly originating from the facial artery trunk, which were relatively thin but multiple (**Figure 2A**). In the other four samples, the facial artery first produced several thicker trunks and then spread in a dendritic form (**Figure 2B**). The median maximum pedicle length was 106 (71.1–142) mm, which qualified the flap for the contralateral reconstruction.

**Table 1:** The branch and pedicle measurements of the facial artery.

Sample	Buccal Branch		Masseter Branch		Maximum pedicle length (mm)
	Quantity	Diameter (mm)	Quantity	Diameter (mm)	
1	4	0.61 (0.36–0.94)	1	1.13	109 (96–120)
2	4	0.61 (0.50–0.94)	1	0.90	89.5 (74.5–113.5)
3	2	0.54 (0.51–0.57)	1	1.20	77.60 (77.1–84.1)
4	7	0.50 (0.50–0.90)	1	0.50	106 (75–142)
5	7	0.50 (0.40–1.50)	1	0.50	110 (99.5–141.5)
6	4	0.65 (0.50–0.70)	1	0.90	115 (104.5–117.5)
7	7	0.42 (0.20–0.62)	1	0.98	120.98 (103.58–139.95)
Median	4	0.50 (0.20–1.50)	1	0.90 (0.50–1.20)	106 (71.1–142)

Data are given as median (range).

The venous drainage of this flap was diverse and mainly through three veins. In half of the patients (12/24), the facial vein joined the common facial vein and drained through the internal jugular vein. In nine of them, the facial vein went posteriorly oblique and joined the external jugular vein. In only three of the 24 patients, the facial vein went anteriorly oblique and joined the anterior jugular vein. There were abundant communicating branches between the cervical veins.

The marginal branch of the facial nerve runs approximately 10 mm above the mandibular margin. Three patterns of the marginal nerves and facial blood vessels were identified. In type I (18/24), the marginal nerve superficially crossed the facial blood vessels (**Figure 2C**); in Type II (2/24), the nerve branches circled both the facial artery and vein (**Figure 2D**); and in Type III (4/24), the marginal nerve circled only one of the pedicle vessels and was trapped between them (**Figure 2E**). The type I pattern allows the pedicle and flap to pass beneath the nerve. In Type II, the flap is passed through the nerve loop. In type III, the artery was cut and re-anastomosed to free the nerves.

### 3.2 Safety for T_2_–T_3_ oncologic defect reconstructions

The enrolled patients were diagnosed with T_2_ **–**T_3_ OSCC (18, 3, and 3 at the tongue, buccal mucosa, and FOM, respectively) (**Table 2**). After tumor resection, the subsequent defects ranged from 30 × 30 to 70 × 50 mm, all of which were immediately reconstructed with C-FAMMIFs. All flaps survived without complications except for one flap that was partially trimmed due to infection. All patients underwent selective neck dissection (SND), including levels I, II, and III, among whom 10 were found to have positive lymph nodes mainly distributed at levels I and II. Adjuvant radiotherapy was administrated after surgery for 13 patients.

**Table 2:** Patient characteristics of the C-FAMMIF group.

Number	Sex	Age	Tumor site	pStage	DOI (mm)	Pathology	Defect size (cm^2^)	Neck dissection	RT	Follow-up months
**1**	M	62	Right FOM	pT_2_N_2b_M_0_	7	SCC	6*4	Right SND	Yes	39
**2**	F	67	Left tongue	pT_3_N_1_M_0_	13	SCC	5.5*3.5	Left SND	Yes	34
**3**	M	39	Right tongue	pT_3_N_0_M_0_	10	SCC	5*3	Right SND	No	33
**4**	M	29	Left tongue	pT_2_N_0_M_0_	7	SCC	5.5*3	Left SND	No	32
**5**	M	35	Left tongue	pT_3_N_1_M_0_	12	SCC	5*3	Left SND	Yes	32
**6**	M	28	Right tongue	pT_3_N_0_M_0_	11	SCC	5*3	Right SND	No	32
**7**	F	41	Left tongue	pT_2_N_0_M_0_	8	SCC	4*3	Left SND	No	31
**8**	M	70	Right buccal mucosa	pT_3_N_0_M_0_	10	SCC	6*4.5	Right SND	Yes	30
**9**	F	62	Right tongue	pT_3_N_0_M_0_	10	SCC	5*3	Right SND	No	29
**10**	M	52	Right tongue	pT_2_N_0_M_0_	7	SCC	5*3.5	Right SND	Yes	28
**11**	M	40	Right tongue	pT_2_N_0_M_0_	7	SCC	5*3	Right SND	No	28
**12**	F	57	Right tongue	pT_2_N_1_M_0_	5	SCC	5.5*3.5	Right SND	Yes	28
**13**	M	67	Left tongue	pT_2_N_1_M_0_	5	SCC	5.5*3.5	Left SND	Yes	27
**14**	F	62	Right buccal mucosa	pT_2_N_2b_M_0_	4	SCC	7*5	Right SND	Yes	27
**15**	M	55	Left tongue tip	pT_3_N_0_M_0_	5	SCC	4*3	Bilateral SND	No	26
**16**	M	53	Right FOM near midline	pT_2_N_0_M_0_	5	SCC	6*4	Biliteral SND	Yes	24
**17**	M	45	Left tongue	pT_2_N_1_M_0_	11	SCC	5*4	Bilateral SND	Yes	24
**18**	M	60	Bilateral FOM	pT_2_N_0_M_0_	2	SCC	6*3	Bilateral SND	No	23
**19**	M	63	Right buccal mucosa	pT_2_N_0_M_0_	9	SCC	6*4	Right SND	No	23
**20**	M	69	Left tongue	pT_3_N_0_M_0_	12	SCC	6*5	Left SND	No	22
**21**	F	68	Right tongue base	pT_3_N_1_M_0_	12	SCC	5*4	Right SND	Yes	22
**22**	F	56	Right tongue	pT_2_N_0_M_0_	2	SCC	3*3	Right SND	No	15
**23**	M	33	Left tongue	pT_2_N_1_M_0_	5	SCC	6*4	Left SND	Yes	14
**24**	M	62	Left tongue	pT_2_N_3b_M_0_	7	SCC	6*5	Left SND	Yes	13

M, male; F, female; DOI, depth of invasion; RT, radiotherapy; FOM, floor of mouth; SCC, squamous cell carcinoma; SND, selective neck dissection.

During follow-up (median 27.5 months), two patients had a locoregional recurrence. One patient was a 62-year-old female (no.9 in **Table 2**) with T3 tongue SCC who underwent tumor resection and unilateral SND. No positive lymph nodes were detected. Ten months later, a neoplasm appeared in the ipsilateral parotid gland and was confirmed to be an SCC metastasis. The other patient was a 53-year-old male (no.16 in **Table 2**) whose primary tumor was located at the right FOM close to the midline and underwent tumor resection and bilateral SND. The pathological examination revealed no positive lymph nodes. After 4 months, the tumor relapsed at the submental region, whereas the FOM mucosa was intact. Neither of the tumor relapses was considered to be associated with flap transfer.

**Figure 3:**
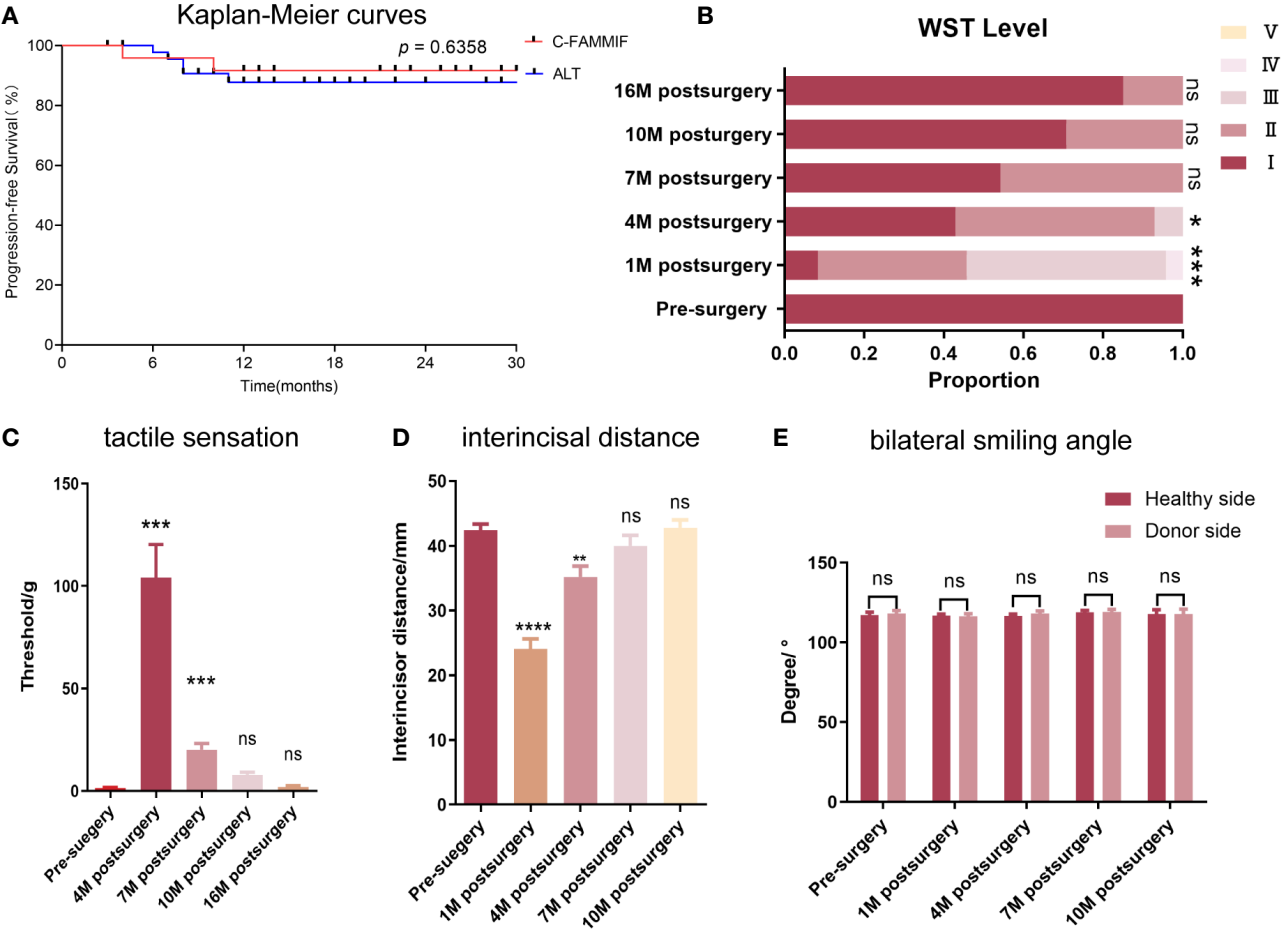
Survival, functional and donor site outcomes. **(A)** the Kaplan-Meier curve of the PFS of the C-FAMMIF and ALT group; **(B, C)** the results of swallowing and tactile sensation assessment. **(B)** most patients’ swallowing function recovered to grade I 16 months after surgery. **(C)** the numbness was severest 4 months after surgery, but gained gradual recovery 16months after surgery. **(D, E)** the results of mouth opening and facial symmetry assessment. **(D)** temporary mouth opening limitation was observed, but the interincisal distance recovered to the presurgical level 10 months after surgery. **(E)** No facial asymmetry was observed before and after the surgery. “**”, “***”, “****” indicate significant difference with *p* < 0.01, < 0.001, < 0.0001, respectively; “ns” indicates no significant difference.

The matched ALT group consisted of 47 patients whose baseline information is shown in **Table 3**. The propensity score distribution plot showed an almost complete overlap after matching (**Supplementary Figure 2**), and no significant differences in baseline information were identified (**Table 3**). The 2-year PFS rate between the two groups was not significantly different (84.6% and 88.2% in the ALT and C-FAMMIF groups, respectively, *p* = 0.6358) (**Figure 3A**).

**Table 3:** The baseline information of the C-FAMMIF and matched ALT group.

Variables	Total No. of patients (%) (n=71)	No. of patients (%)		*p* value
		C-BMMIF (n=24)	Free flaps (n=47)	
Sex				
M	51 (71.8%)	17 (70.8%)	34 (72.3%)	1.0 0.680
F	20 (28.2%)	7 (29.2%)	13(27.7%)	
***Age***	25~76	28~70	25~76	
Site				
Tongue	54 (76.1%)	18 (75.0%)	36 (76.6%)	0.376
Bucca	12 (16.9%)	3 (12.5%)	9 (19.1%)	
FOM	5(7.0%)	3 (12.5%)	2 (4.3%)	
T stage				
T1	4 (5.6%)	0 (0.0%)	4 (8.5%)	0.139
T2	41 (57.7%)	18 (75.0%)	23 (49.0%)	
T3	25 (35.2%)	6 (25.0%)	19 (40.4%)	
T4	1 (1.4%)	0 (0.0%)	1 (2.1%)	
N stage				
N0	42 (59.2%)	14 (58.3%)	28 (59.6%)	0.910
N1	15 (21.1%)	5 (20.8%)	10 (21.3%)	
N2	10 (14.1%)	3 (12.5%)	7 (14.9%)	
N3	4 (5.6%)	2 (8.4%)	2 (4.2%)	
M stage				
M0	71 (100%)	24 (100%)	47 (100%)	1.0
SND				
No SND	1 (1.4%)	0 (0.0%)	1 (2.1%)	0.604
Unilateral	65 (91.5%)	23 (95.8%)	42 (89.4%)	
Bilateral	5 (7.1%)	1 (4.2%)	4 (8.5%)	
Adjuvant RT				
Yes	32 (45.1%)	13 (54.2%)	19 (40.4%)	0.271
No	39 (54.9%)	11 (45.8%)	28 (59.6%)	
Smoking				
Yes	42 (59.2%)	14 (58.3%)	28 (59.6%)	0.920
No	29 (40.8%)	10 (41.7%)	19 (40.4%)	
Alcohol consumption				
Yes	22 (31%)	11 (45.8%)	11 (23.4%)	0.053
No	49 (69%)	13 (54.2%)	36 (76.6%)	
***Total***	71	24	47	

M, male; F, female; FOM, floor of mouth; SND, selective neck dissection; RT, radiotherapy.

### 3.3 Functional outcomes and donor site morbidity

**Figure 4:**
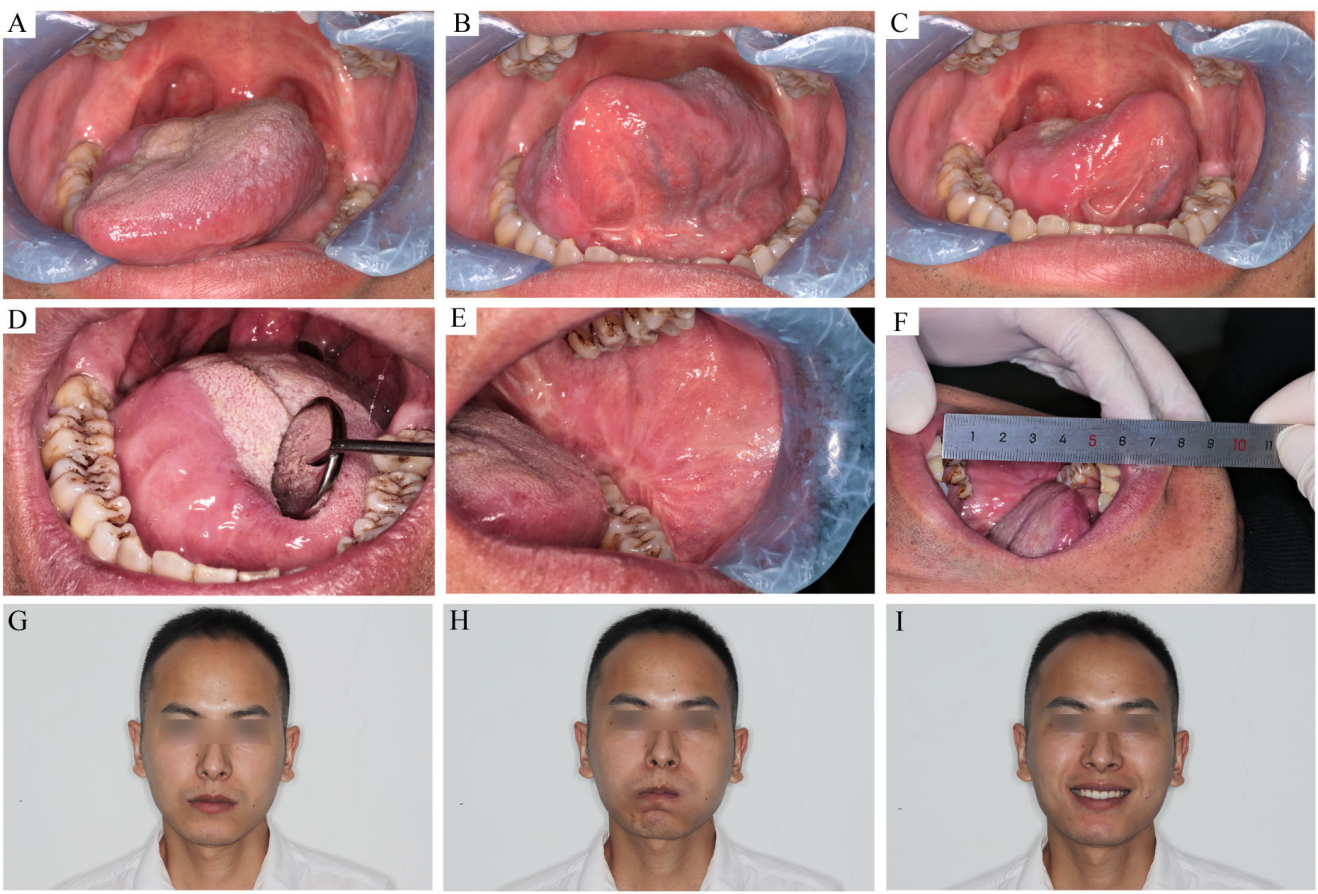
The recipient and donor site conditions 7 month after the surgery. **(A–C)** the tongue movement range after T_3_ tongue SCC resection and C-FAMMIF reconstruction. **(D)** the flap merged with the residual tongue harmoniously. **(E)** the donor site healed primarily **(F)**: the interincisal distance was good. **(G–I)** no facial asymmetry was present.

The main postoperative symptoms were dysphagia, tongue inflexibility, and dysesthesia. Dysphagia was obvious 1 month after surgery, especially in patients whose tumors were located at the base of the tongue. One patient had grade IV dysphagia, and 12 patients had grade III. Prominent recovery was observed after 6 months (**Figure 3B**) when all patients could finish swallowing 30 ml of water without choking (levels I–II). After the wound healed, the flap mucosa merged seamlessly with the adjacent tissue (**Figure 4D**). Only a moderate scar formed at the flap junction and no obvious sclerosis or shrinkage of the flap mucosa was observed. The flap was pliable to allow considerable flexibility of the residual tongue (**Figures 4A–C**). Recovery of sensation was slow but notable. None of the patients could feel even the thickest filament (>100 g) 1 month after the surgery; however, after 3 months, all patients had a rough mechanical sensation to feel the 104.13 ± 16.15 g pressure and gradually recovered to be able to sense the 1.95 ± 0.68 g pressure throughout the 16-month follow-up (**Figure 3C**).

Facial nerve palsy and trismus were the most common complications associated with this flap. A month after surgery, 13 patients had dynamic mouth asymmetry but maintained intact oral function and static mouth symmetry (H-B grade 4). After 1 year, only a mild weakness in the perioral muscles (H-B grade 2) was observed in three patients, and the remaining 21 patients gained both static and dynamic mouth symmetry (**Figures 4G–I**). The smile angle analysis showed no significant differences throughout the follow-up period (**Figure 3E**). All donor sites healed completely, with the buccal fat pad undergoing mucosal transformation and moderate scarring (**Figure 4E**). The average interincisal distance was 24 ± 1.49 mm 1 month after surgery, which gradually increased to 40.26 ± 1.61 mm after 6 months and 42.7 ± 1.23 mm after 1 year, almost to the presurgical level (**Figures 3D**, **4F**). Adjuvant therapy did not worsen the mouth opening significantly; the interincisal distance of the thirteen patients who received adjuvant radiotherapy was 41.2 ± 1.57 mm 1 year after the surgery.

## 4 Discussion

To our knowledge, this study is the first to develop the concept of C-FAMMIF, validate its anatomical basis, and assess its clinical application, including oncological safety, functional outcomes, and donor site morbidity.

Facial-artery-based regional flaps played an indispensable role in oral defect reconstruction because of their proximity, especially when microvascular anastomosis was unavailable (21). However, the lymph node metastasis in level I made it controversial to employ facial-artery-based regional flaps in oncologic defect reconstruction (18, 19). Strict limitation to the cN_0_ patients and pedicle skeletonization were suggested in previous studies (18, 20, 22, 23), but occult metastasis was still present in 34%–45% of cN_0_ necks (19, 24, 25), and 25% of the skeletonized flap pedicles were histologically confirmed to contain lymph nodes (26). To overcome this difficulty, contralateral facial-artery-based nasolabial flap and submental island flap were introduced in recent years (27, 28). The FAMMIF has been practiced for more than three decades and exhibited favorable functional and aesthetic outcomes with its featured mucosa-to-mucosa restoration (12, 14, 29, 30). Our study investigated the feasibility of C-FAMMIF for validating its application in oral oncologic defect reconstruction. In this study, 41.7% of patients were pathologically confirmed to have cervical lymph nodes metastasis, and no flap-associated relapse (relapses near the flap or along the pedicle) was observed after > 2 years follow-up. C-FAMMIF was as safe as ALT in ipsilateral pN+ patients.

The anatomy of the facial artery has been investigated in earlier studies. It supplied FAMMIF with rich buccal branches (14), which was confirmed in our study. Moreover, we provided detailed measurements of the buccal branches and their distances from the origin of the facial artery, reflecting the maximum allowable pedicle length, which was the basis for contralateral reconstruction. Free FAMMIF for contralateral buccal defect reconstruction has been reported previously (31); however, our results demonstrate that cutting and anastomosing the blood vessels is unnecessary since adequate pedicle length could be obtained. Masseter muscle flaps have been used in oral defect reconstruction since 1978 (32, 33). Our study found that it is practical to include a masseter muscle island in C-FAMMIF for larger defects. The marginal branch of the facial nerve intercrosses with the facial blood vessels and should receive additional attention during all facial-artery-based flap harvests. Previous anatomical studies have unveiled its approximate course, relative position to the mandibular margin, and piercing position of the cervical deep facia (34–36). From the perspective of flap harvest, its dimensional relationship with the facial blood vessels was more concerning but lacking in former studies. Our study categorized nerve-pedicle relationship patterns into three categories. The majority had the nerve running superficially to the blood vessels; however, the circling of one or both blood vessels occurred in one-quarter of the patients, where the nerve was likely to be injured if the surgeon was unaware of the special pattern.

FAMMIF is believed to have a very limited tissue amount and thickness. However, with the inclusion of an arterialized masseter muscle island, the T_3_ oncologic defect can be easily restored. In our study, the largest tongue defect measured 50 × 60 mm in area and was close to the septum in depth; thus, a masseter muscle island measuring 30 × 30 mm was included to fill the defect space, and the buccinator myomucosal island restored the defected mucosa. In T_2_–T_3_ buccal mucosa or FOM reconstruction, the flap area was more important than the thickness. Adequate areas create space for tongue mobility and mouth opening. The largest defect area measured 50 × 70 mm and was successfully reconstructed using the buccinator myomucosal island alone.

The buccal fat pad is a special structure located at the space between the buccinator, masseter, and skin. During flap rising, the buccal fat pad was revealed in the posterior boundary of the flap. The size of buccal fat pad was associated with the overall body weight of patient. The buccal fat pad was reported to have a mean volume of 10cm^3^, a thickness of 6mm, and could cover an area of 100 mm^2^ (37). A study showed that the buccal fat pad could successfully close soft tissue defects as large as 70 mm × 50 mm × 20mm (38). In our practice, the largest C-FAMMIF measured 50 mm × 70 mm and the donor site was successfully closed with the buccal fat pad with primary healing. The blood supply of the buccal fad pad mainly depends on the capillary network in the capsule (37), which should be well preserved to ensure the blood supply and to avoid dehiscence. In our practice, iodoform gauze was used to cover and pack the donor site to avoid the direct exposure of buccal fat pad to the oral cavity. Further covering with artificial membrane or skin graft was not necessary. The iodoform gauze were removed 2 weeks after surgery, when the surface of buccal fat pad underwent epithelialization.

Nasogastric tube was applied in all patients in order to maintain oral hygiene and to prevent aspiration. The time of nasogastric feeding was dependent upon the swallowing function of the patient, which was closely related to the size and position of the tumor, and the resection range of the suprahyoid muscles. In our practice, water swallowing test was used to evaluate the swallowing function before the nasogastric tube was removed. When the swallowing function recovered to WST grade I to II, which meant that no choking was present, it was time to transit from nasogastric feeding to oral feeding. If the swallowing function was graded WST grade III to V, which meant choking occurred during swallowing, the tube was retained and the swallow training continued. Since both the C-FAMMIF and the ALT cohort were mainly composed of patients staged T_2_-T_3_, the nasogastric feeding time was similar in both groups.

Based on our experience, we want to highlight several points regarding flap harvesting. First, the contralateral flap harvesting procedure should be independent of tumor resection and ipsilateral neck dissection. The surgical sites did not communicate, and the surgical sheets and instruments were changed between procedures. Second, surgeons should be aware of the diverse venous drainage paths. Drainage failure was considered to be the main drawback of FAMMIF (39). In 50% of our cases, the vein drained through the superficial veins (the external and anterior jugular veins), which were likely to be damaged during neck dissection. Thus, harvesting the flap from the contralateral side is safer. Third, the donor site should be carefully managed to prevent salivary fistulae, infections, and trismus. Compression and nasogastric feeding were important during the first 2 weeks after surgery. Instrument-assisted mouth-opening training should be introduced when the fat pad has healed, and scars begin to form. Adjuvant radiotherapy does not worsen mouth opening if proper postsurgical care and exercise are provided.

The flap mucosa showed early and rapid tactile recovery. Sensation rehabilitation of the grafted tissue is difficult because the nerve endings in the flaps are interrupted with the recipient site. Innervated flaps were introduced to recover the flap sensation. The non-innervated FAMMIF showed a faster and sharper sensation recovery than the innervated skin flaps (40, 41), which is consistent with other studies on myomucosal flaps. There are several explanations for this. First, more nerve endings were present in the mucosa than in the skin. Second, reinnervation is easier to establish in the same tissue type. Regaining temperature and pain were expected from further observations (15, 29).

Our study reveals the vast application potential of C-FAMMIF in oral oncologic defect reconstruction, particularly in patients with ipsilateral radiotherapy, a history of neck surgery, or ipsilateral clinically positive lymph nodes. However, this study has limitation. It was a retrospective study with a small sample size and a short follow-up period. Hence, prospective randomized controlled trials with larger sample sizes are needed to compare oncologic safety and functional outcomes.

## 5 Conclusion

The C-FAMMIF has stable anatomical basis and is a reliable and safe option for T_2_-T_3_ oral oncologic defect reconstruction, particularly in patients with ipsilateral radiotherapy, a history of neck surgery, or ipsilateral clinically positive lymph nodes.

## Data availability statement

The original contributions presented in the study are included in the article/**Supplementary Material**. Further inquiries can be directed to the corresponding author.

## Ethics statement

The studies involving humans were approved by Ethics committee of Guanghua School of Stomatology, Sun Yat-sen University. The studies were conducted in accordance with the local legislation and institutional requirements. The participants provided their written informed consent to participate in this study. Written informed consent was obtained from the individual(s) for the publication of any potentially identifiable images or data included in this article. Written informed consent has been obtained from the individual whose image was used in the Supplementary materials. The image was original and not from any existing publication.

## Author contributions

MH: Data curation, Writing – review & editing. PL: Formal analysis, Writing – original draft. LY: Writing – review & editing. YX: Writing – review & editing. LZ: Data curation, Writing – review & editing. YS: Methodology, Validation, Writing – review & editing. YL: Methodology, Validation, Writing – review & editing. GZ: Methodology, Writing – review & editing. GL: Conceptualization, Supervision, Writing – review & editing. SZ: Conceptualization, Funding acquisition, Supervision, Writing – review & editing.
