# Supplementary material for: Contralateral facial artery myomucosal island flap for the reconstruction of T2-T3 oncologic oral defects
Source: Front Oncol. 2024 Jun 4;14:1393687. doi: 10.3389/fonc.2024.1393687 (PMC11184065; doi:10.3389/fonc.2024.1393687)

**Supplementary Table 1** House-Brackman facial paralysis scale

| Grade | Description | Gross function | Resting appearance | Dynamic appearance |
| --- | --- | --- | --- | --- |
| Ⅰ | Normal | Normal | Normal | Normal |
| Ⅱ | Mild dysfunction | Slight weakness with effort, may have mild synkinesis | Normal | Mild oral and forehead asymmetry; complete eye closure with minimal effort |
| Ⅲ | Moderate dysfunction | Obvious asymmetry with movement, noticeable synkinesis or contracture | Normal | Mild oral asymmetry, complete eye closure with effort, slight forehead movement |
| Ⅳ | Moderately severe dysfunction | Obvious asymmetry, disfiguring asymmetry | Normal | Asymmetrical mouth, incomplete eye closure, no forehead movement |
| Ⅴ | Severe dysfunction | Barely perceptible movement | Asymmetric | Slight oral/nasal movement with effort, incomplete eye closure |
| Ⅵ | Total paralysis | None | Asymmetric | No movement |

**Supplementary Figure 1** Measurements of the smile angles


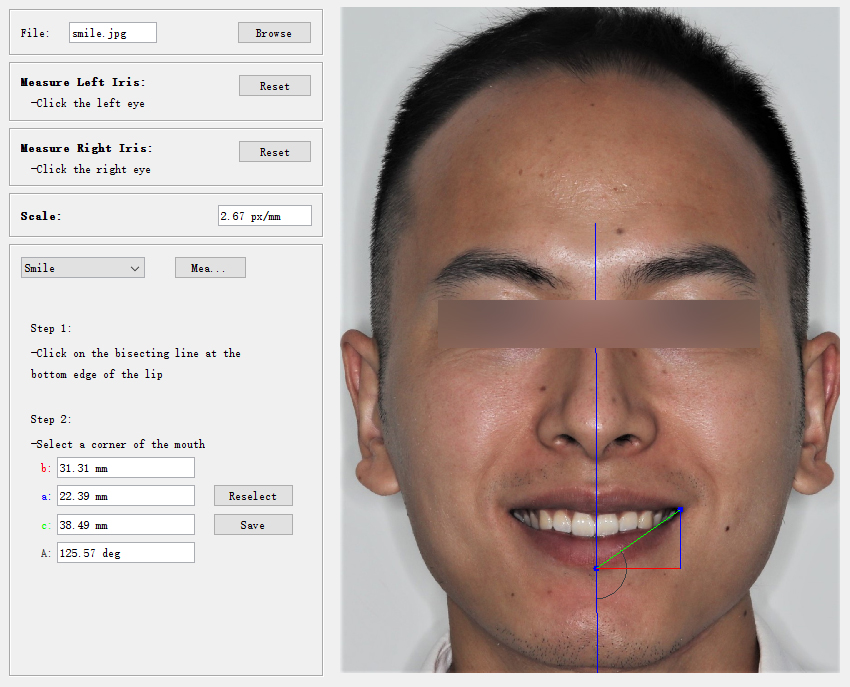


**Supplementary Figure 2** The propensity score distribution plots

a: before the matching; b: after the matching.


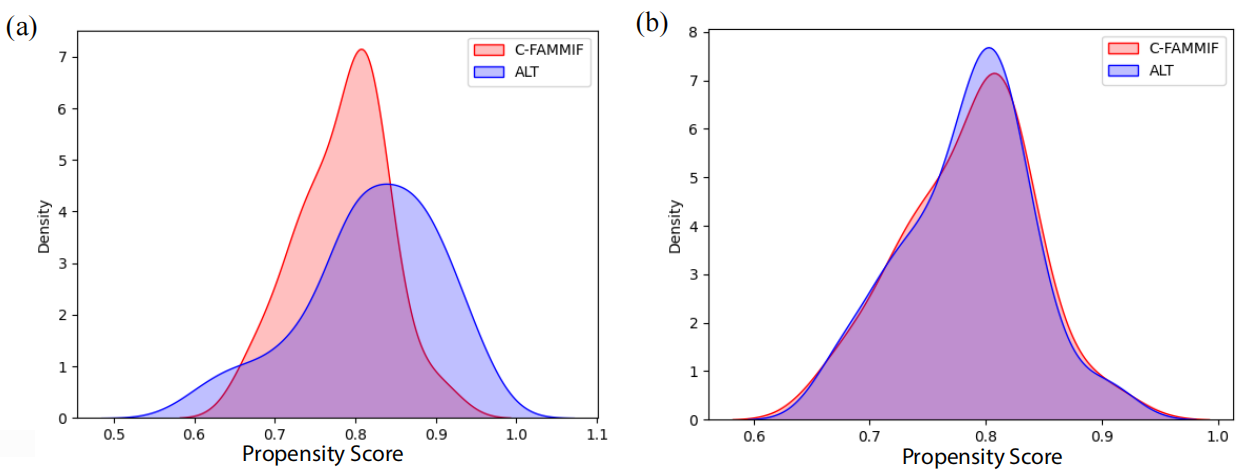

Supplement: Supplementary file 1 [file DataSheet_1.docx]
